# Supplementary material for: Surface Modification of Additively Fabricated Titanium-Based Implants by Means of Bioactive Micro-Arc Oxidation Coatings for Bone Replacement
Source: J Funct Biomater. 2022 Dec 8;13(4):285. doi: 10.3390/jfb13040285 (PMC9781821; doi:10.3390/jfb13040285)
Supplement: Supplementary file 1 [file jfb-13-00285-s001.zip › jfb-2041482-supplementary.pdf]

Surface Modification of Additively Fabricated Titanium-Based Implants by Means of Bioactive Micro-Arc Oxidation Coatings for Bone Replacement

Anna I. Kozelskaya [2], Sven Rutkowski [2,*], Johannes Frueh [1,2,*], Aleksey S. Gogolev [2], Sergei G. Chistyakov [2], Sergey V. Gnedenkov [3], Sergey L. Sinebryukhov [3], Andreas Frueh [2], Vladimir S. Egorkin [3], Evgeny L. Choynzonov [4], Mikhail Buldakov [4], Denis E. Kulbakin [4], Evgeny N. Bolbasov [2], Anton P. Gryaznov [2], Ksenia N. Verzunova [2], Margarita D. Apostolova [5] and Sergei I. Tverdokhlebov [2,*]

[1] Faculty of Medicine and Health, Harbin Institute of Technology, Harbin 150080, China
[2] School of Nuclear Science & Engineering, Tomsk Polytechnic University, 30 Lenin Avenue, 634050 Tomsk, Russia
[3] Institute of Chemistry of FEB RAS, 159 Pr. 100-letiya Vladivostoka, 690022 Vladivostok, Russia
[4] Cancer Research Institute, Tomsk National Research Medical Center, Russian Academy of Sciences, 5 Per. Kooperativny, 634050 Tomsk, Russia
[5] Roumen Tsanev Institute of Molecular Biology, Bulgarian Academy of Sciences, Acad. G. Bonchev Str., bl. 21, 1113 Sofia, Bulgaria
* Correspondence: rutkowski_s@tpu.ru (S.R.); johannes.frueh@alumni.ethz.ch (J.F.); tverd@tpu.ru (S.I.T.)

Supplementary Materials and Methods

MAO coating fabrication on the surface of flat disk-like samples: The preparation of the sample surfaces before the coating included the chemical etching in an aqueous solution of nitric and hydrofluoric acids in volume ratios of HNO_3:HF:H_2O = 1:2.5:2.5, at room temperature for 10 – 15 s, followed by a neutralization in a 1% aqueous solution of sodium hydroxide for 15 s and the three-time rinsing in distilled water, where the beaker was placed in an ultrasound bath.

To fabricate the first MAO coating (MAO 1), the disk-like samples were processed in a water-based electrolyte solution with 10% phosphoric acid (H_3PO_4, reagent grade, Component-Reaktiv, LLC, Moscow, Russian Federation) at a voltage of 270 V, a voltage rise rate of 2 V/s, a pulse repetition rate of 100 Hz and a pulse duration of 70 s for 15 minutes. A supersaturated solution of CaO (reagent grade, Component-Reaktiv, LLC, Moscow, Russian Federation) in a 10% H_3PO_4 solution with 10 g/L dispersed hydroxyapatite (reagent grade, Fluidinova, Maia, Portugal) was applied to form the second MAO coatings (MAO 2) on the disk-like samples. The MAO process parameters were as follows: voltage 320 V, voltage rise rate: 3 V/s, a pulse repetition frequency of 200 Hz, a pulse duration of 100 s and a formation time of 15 minutes. To form the third MAO coating (MAO 3) on the disk-like samples, an electrolyte solution containing calcium acetate hydrate (reagent grade, $Ca(CH_3COO)_2 \cdot H_2O$, c = 15.6 g/L, Component-Reaktiv, LLC, Moscow, Russian Federation) and disodium hydrogen phosphate anhydrous (reagent grade, Na_2HPO_4, c = 36.8 g/L, VEKTON JSC, Saint Petersburg, Russian Federation), with the addition of H_3PO_4 at a concentration of 2.5 mL/L was applied. The formations of MAO 3 coatings were performed at a voltage of 500 V, a voltage rise rate of 3 V/s, a pulse repetition frequency of 500 Hz and a pulse duration of 400 s for 15 minutes. In all cases for coating the disk-like samples, the electrolyte solution temperature was kept at 15 °C during the MAO process.

MAO coating fabrication on the surface of 3D printed implant samples: Due to the complex geometric structure of 3D printed samples (SI Figure S1 and S2C), the parameters of the MAO process for coating their surfaces differ from parameters for the flat disk-like samples. Therefore, the coatings of the MAO 1 were treated in an electrolyte containing 10% H_3PO_4 (reagent grade, Component-Reaktiv, LLC, Moscow, Russian Federation) at a voltage of 320 V with a voltage rise rate of 3 V/s, with a pulse repetition rate of 230 Hz and a pulse duration of 120 s for 50 minutes. The coatings of the MAO 2 were fabricated in a supersaturated solution of CaO (purchased from Component-Reaktiv, LLC, Moscow, Russian Federation) in 10% H_3PO_4 with 10 g/L dispersed hydroxyapatite (purchased from Fluidinova, Maia, Portugal) at process parameters: a voltage of 320 V; a voltage rise rate of 3 V/s, a pulse repetition rate of 230 Hz; pulse duration of 120 s; formation time – 50 minutes. And at last, the micro-arc oxidation process of the samples of MAO 3 was carried out in an electrolyte containing calcium acetate hydrate ($Ca(CH_3COO)_2 \cdot H_2O$, c = 15.6 g/L, purchased from Component-Reaktiv, LLC, Moscow, Russian Federation) and disodium hydrogen phosphate anhydrous (Na_2HPO_4, c = 36.8 g/L, purchased from VEKTON, JSC, St. Petersburg, Russia), with the addition of H_3PO_4 at concentrations of 2.5 mL/L at follows parameters: a voltage of 500 V; a voltage rise rate of 3 V/s, a pulse repetition rate of 500 Hz and a pulse duration of 400 s; formation time – 20 minutes. The electrolyte temperature in all cases did not exceed 25 °C during the MAO process.

Supplementary Results and Discussion

X-ray diffraction: X-ray patterns of MAO 1 coating display reflections from the planes (101), (004), and (200) at the angles 2 = 25.26°, 37.60°, 48.12° and which are correlating to the anatase phase (Figure 3D). In turn, the reflections from the planes of (110), (101), (200), (111), (211), (220), (002) and (301) at the angles of 2 = 27.32°, 36.04°, 39.58°, 41.18°, 54.26°, 56.63°, 62.92° and 68.82°, respectively correspond to rutile phase. The remaining peaks present in the diffraction pattern of the MAO 1 coatings correspond to reflections of the α-Ti phase, which are the reflections from the planes of (100), (002), (101), (102), (110), (103), (200), (112) and (201) at the angles of 2 = 35.40°, 38.46°, 40.38°, 53.30°, 63.68°, 71.02°, 75.18°, 76.98° and 78.28°, respectively. The phase fractions of the crystallite phases reveals that the amorphous phase is the dominating fraction followed by α-Ti, rutile and anatase (Figure 3E). The large excess of amorphous phase suggests that a high level of biocompatibility can be expected (Figure 3D, 3E, SI Figure S5 and SI Table S4 – S6). It is likely that the amorphous phase of MAO 2 and MAO 3 is predominantly calcium

phosphate, while the one for MAO 1 is amorphous titanium dioxide. A possible reason for the absence of rutile and anatase phases in MAO 2 coatings is their great thickness, because of the formation of titanium dioxide occurs predominantly on the surface of the metal substrate at the beginning of the MAO process. The thicker the coating on the metal surface, the less likely titanium dioxide phases can be detected.

The X-ray patterns of the MAO 3 coatings look very similar to the ones of the MAO 1 coatings (Figure 3D). Intensity and full width at half maximum of the crystallite peaks for the anatase phase are larger compared to the same peaks in the X-ray diffraction patterns of the MAO 1 coatings, hinting either a more regular phase, or a larger phase fraction. In addition, some of the peaks at angles 2 = 27.32°, 36.04°, 41.18°, 68.82° correspond to reflections from the (110), (101), (111) and (301) planes of the crystalline phase of rutile are missing on the X-ray patterns of MAO 3 coatings. It should be noted that the distribution of crystallite fraction for this coating is comparable to MAO 1 (Figure 3E, SI Figure S6 and SI Table S4 – S6). It has been reported that the presence of titanium dioxide polymorphs such as rutile and anatase promotes biocompatibility, cell adhesion and proliferation of such type of coatings [1–3].

The X-ray diffraction patterns of the MAO 2 coatings differ significantly from the MAO 1 and MAO 3 coating samples. MAO 2 displays significant diffuse scattering from the quasi-amorphous phase in the 2 range from 17° to 35° and a few weak peaks at angles 2 = 35.46°, 40.48°, 75.10° and 78.34°, which correspond to reflections from (100), (101), (200), and (201) planes of the α-Ti phase. Not even one peak corresponds to the crystalline phase of the calcium phosphate coating, which indicates its highly amorphous structure. It is known, that the amorphous calcium phosphate materials contribute to improving osteoconduction in the early stages (unlike crystalline ones) due to the rapid dissolution and release of calcium and phosphorus [4,5]. The phase composition, elemental composition mapping and crystal lattice parameters for the examined coatings are given in Figure 3E, SI Figure S5, SI Table S3 and S4, respectively.

Scratch Test: Upon higher magnifications of the scratches, micro-cracks are clearly observed, propagating in opposite directions inside the grooves and opening in the direction of the indenter movement (SI Figure S7, top view zoom-in micrographs). The formation of such cracks is caused by the action of tensile stress behind the indenter. It should be noted that the cracks are localized inside the grooves. Damage features by means of chevron cracks for MAO 1 and MAO 3 coatings and by means of arc tensile cracking for MAO 2 coatings (SI Figure S7, top view zoom-in micrographs) indicate on brittle tensile cracking pattern of the investigated coatings. Cracks located outside the groove observed on the MAO 3 coatings were not caused by the action of the indenter but were formed on the surface during the formation of coatings under the action of micro-arc discharges (SI Figure S7C, top view zoom-in micrograph). It is assumed that these cracks are superficial and do not propagate to the entire depth of the coatings, since delamination of the coatings from the substrate, as in the two previous cases, was not observed. For this reason, it can be assumed that the adhesion and mechanical strength of the MAO coatings investigated is sufficient. This statement is supported by the indentation profile curves shown in SI Figure S7. Inside the plow grooves and near to them, crumbling particles of ploughed material are also observed (SI Figure S7, top view zoom-in micrographs).

Supplementary Figures

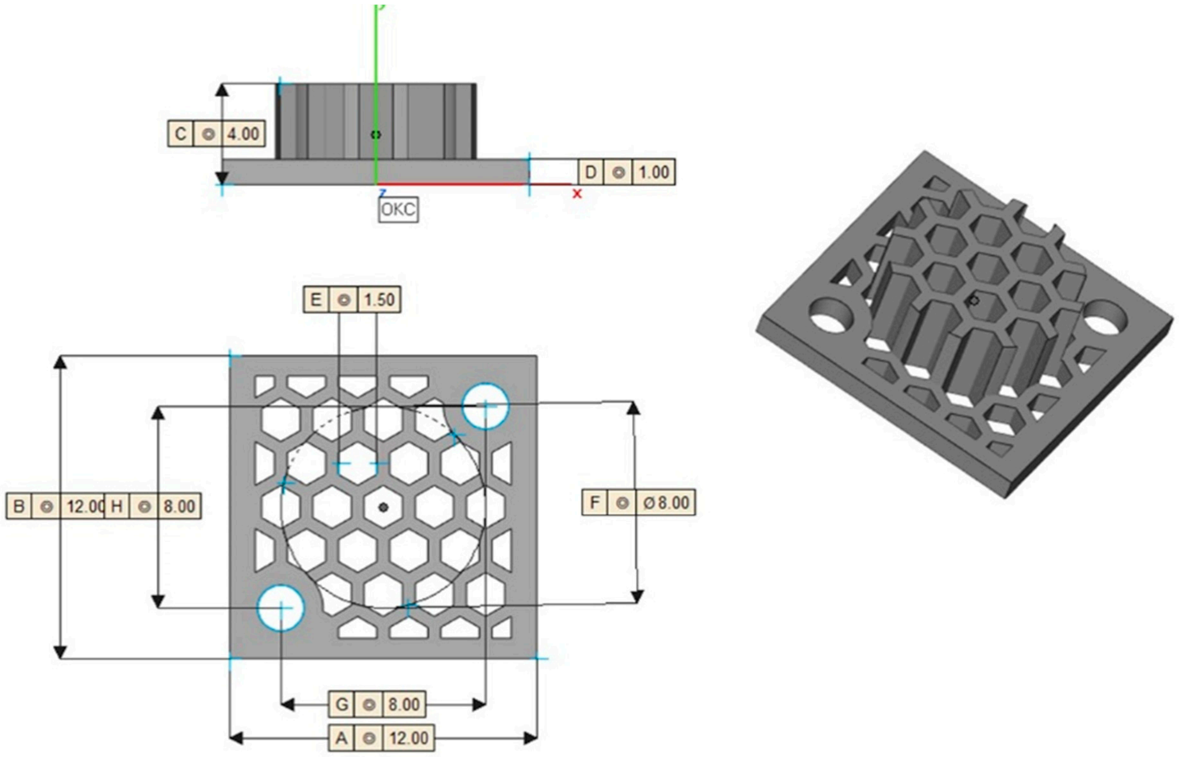

Figure S1. Technical drawings of the 3D printed implant samples with a mushroom-like shape.

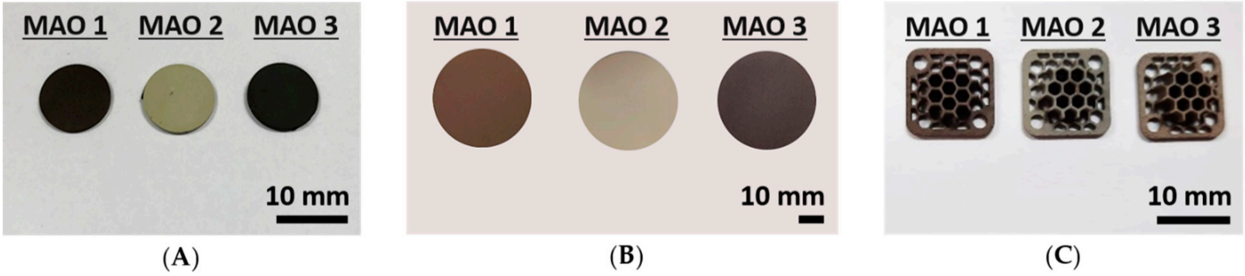

Figure S2. Macroscopic appearance of the sample types utilized in this study with applied MAO coatings. (**A**) Small titanium disks with a diameter of 10 mm, (**B**) Large titanium disks with a diameter of 40 mm and (**C**) 3D printed titanium implant samples with a mushroom-like shape.

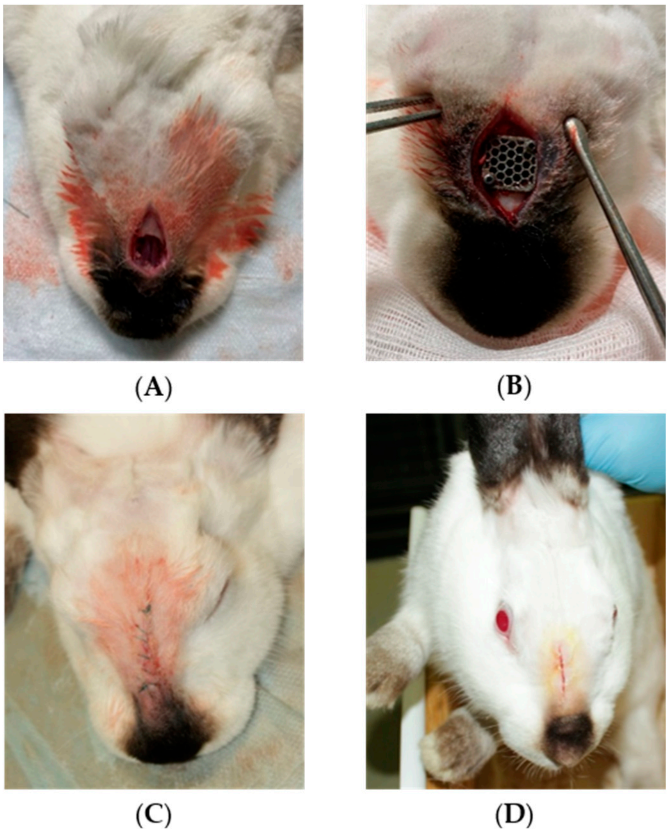

Figure S3. (A) Photographs of the formed bone defect, (**B**) fixation of the 3D printed implant, (**C**) the appearance of the rabbits after suturing the wound (**D**) and after removing the sutures.

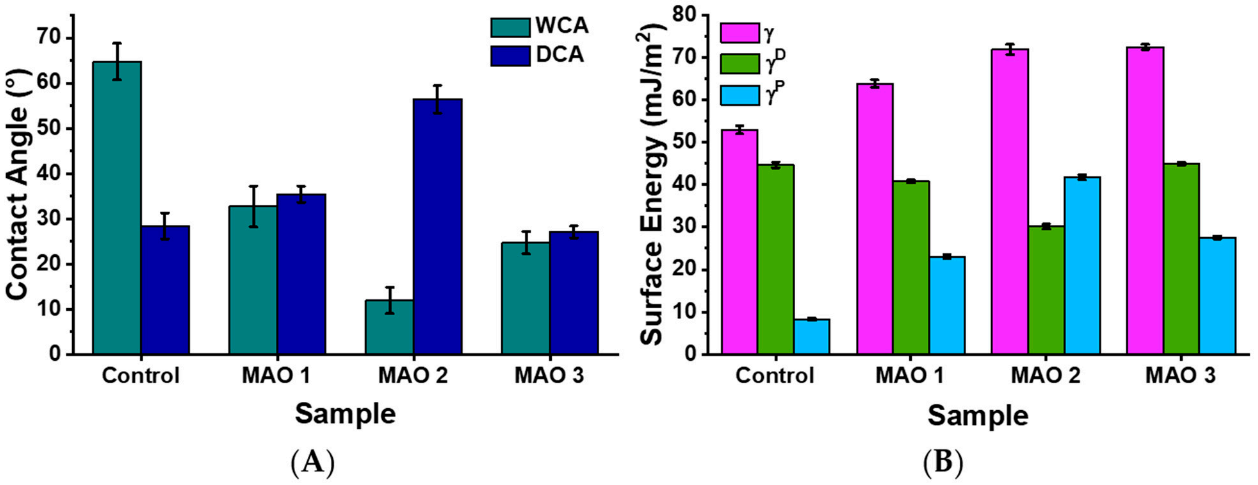

Figure S4. (A) Water contact angle (WCA) and diiodomethane contact angles (DCA) and (B) resultant surface energy , as well as the disperse component ^{D} and the polar component ^{P} of . The values are given in Table S2.

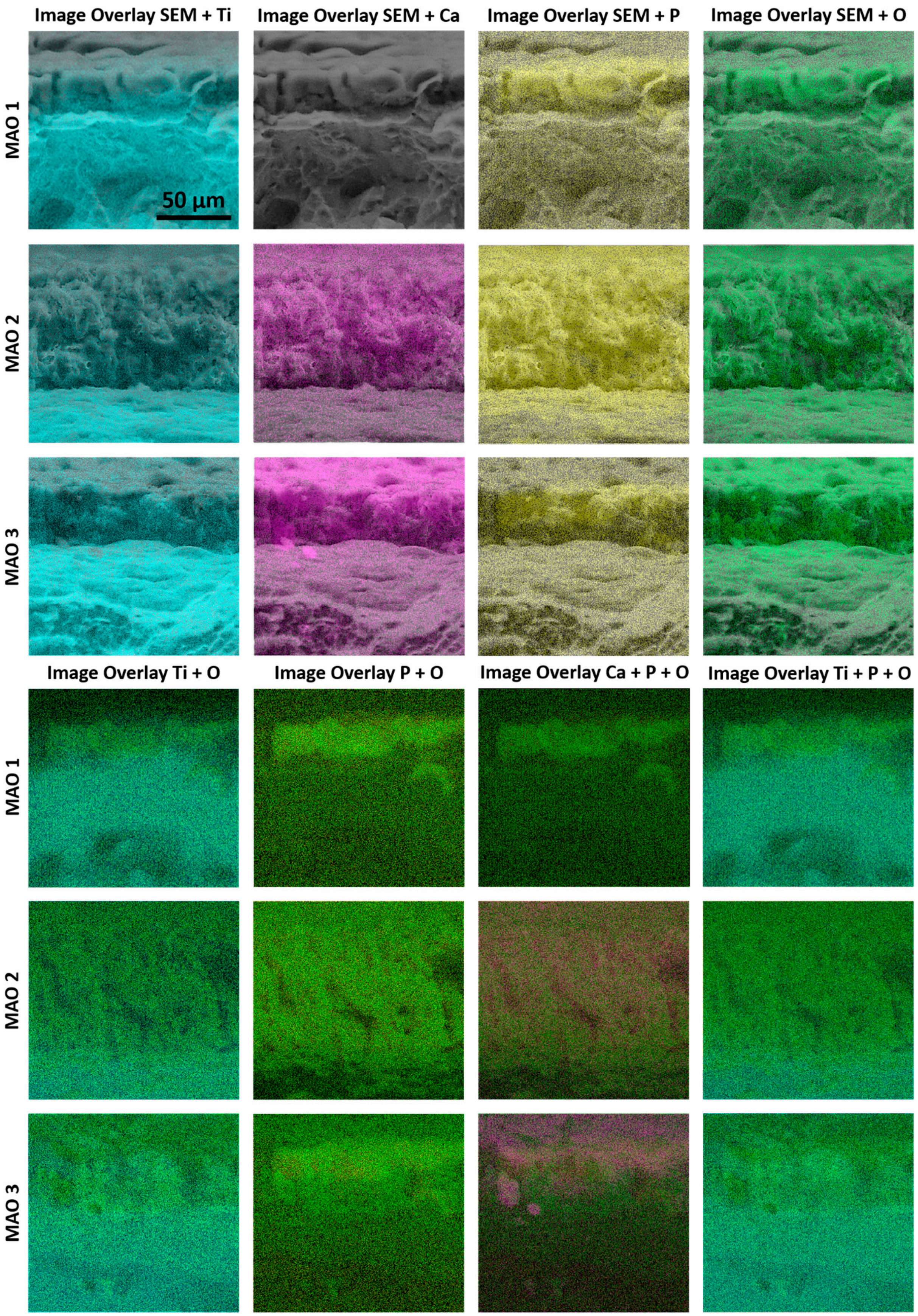

Figure S5. Different image overlays of EDX elemental mapping micrographs of the elements titanium (Ti), oxygen (O), phosphorus (P) and calcium (Ca) for the examined MAO 1 – 3 coatings. The scale bar is 50 mm and representative for all micrographs shown in this figure.

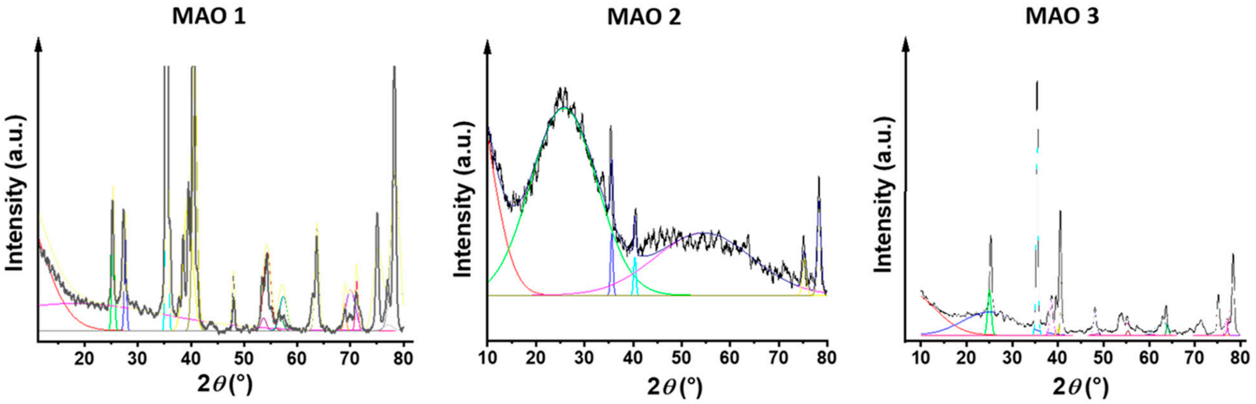

(A) (B) (C)

Figure S6. Gaussian fits of the X-ray scattering patterns shown in Figure 3 of the main manuscript.

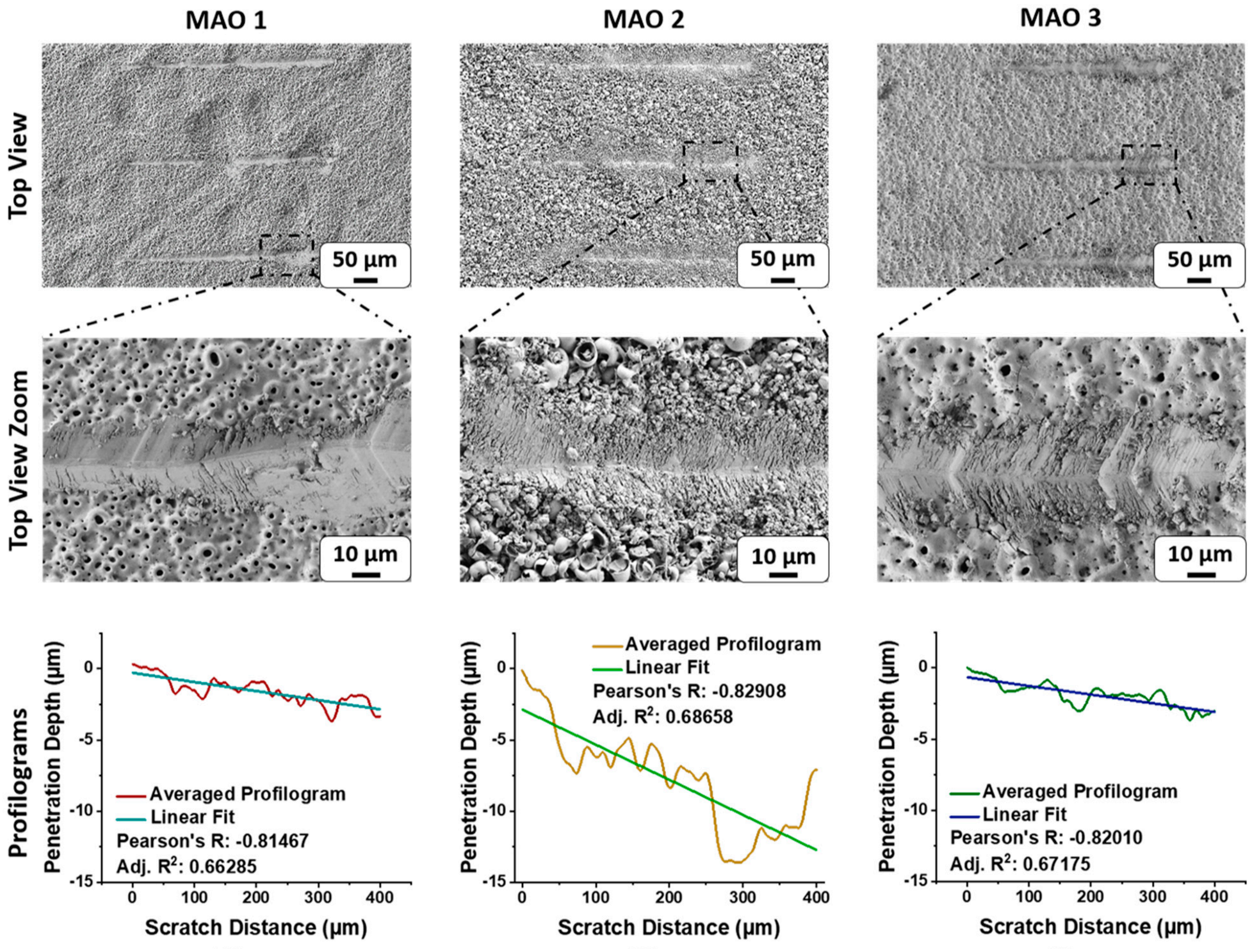

(A) (B) (C)

Figure S7. SEM micrographs of the investigated MAO coating surfaces with top views shown in the upper line and corresponding top view zooms in the lower line. (**A**) MAO 1 coating, (**B**) MAO 2 coating and (**C**) MAO 3 coating after scratch test. The last row displays averaged scratch test profilograms (consists of three scratch test measurements) of the MAO coatings with their respective linear fits included.

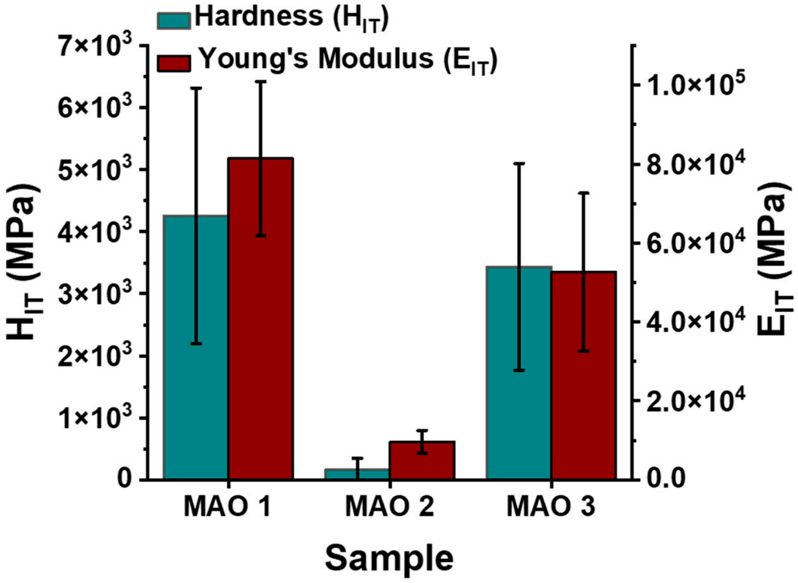

Figure S8. The indentation hardness (H_{IT}) and indentation elastic modulus (E_{IT}) of the coatings MAO 1, MAO 2 and MAO 3, values displayed in Table S8.

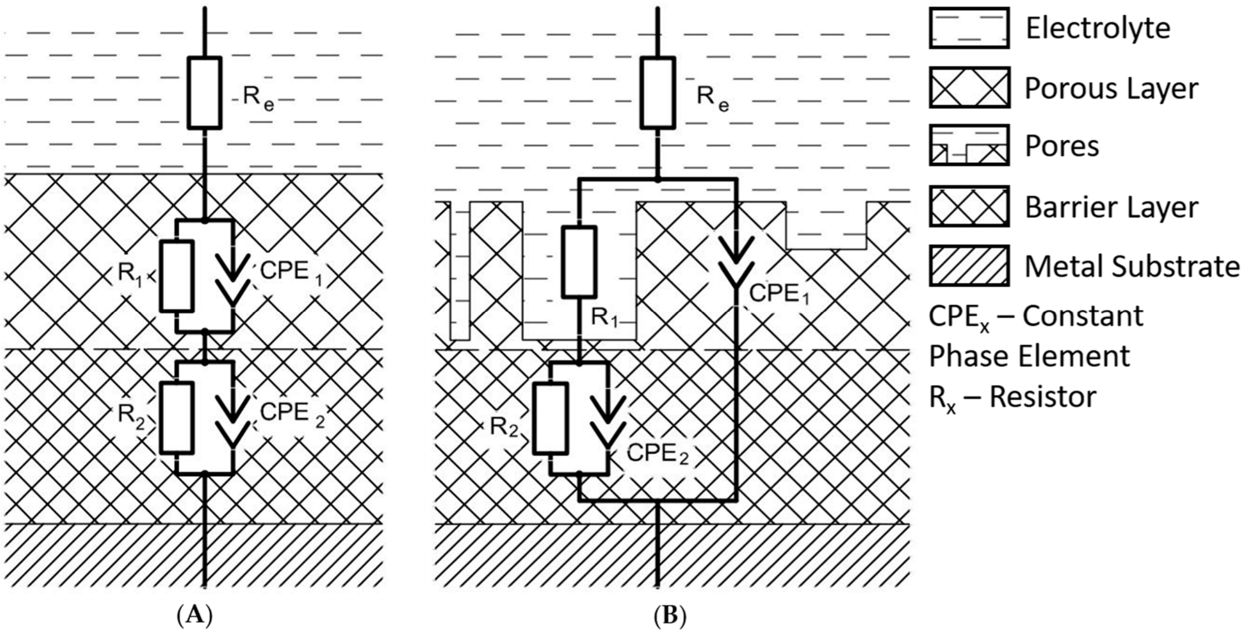

Figure S9. Equivalent electrical circuits schemes used for modelling the electrochemical impedance spectroscopy (EIS) spectra shown in Figure 3F – 3H in the main manuscript for: (**A**) an unmodified titanium alloy sample surface and (**B**) a MAO coated sample surface.

Supplementary Tables

Table S1. Average coating thickness and morphological surface parameters of the investigated MAO coatings. The Roughness is given as average roughness (Ra) and average height difference (Rz).

Sample	Average Coating Thickness (m)	Number of Pores per 1000 m^2	Average Pore Diameter (m)	Roughness (m)	
				Ra	Rz
**MAO 1 (titanium oxide)**	3.90 ± 1.16	34.5 ± 1.5	0.90 ± 0.56	1.7 ± 0.3	4.2 ± 0.4
**MAO 2 (Ca/P ≈ 0.4)**	42.00 ± 2.09	25.6 ± 2.3 (pores) 19.6 ± 2.4 (spheres)	3.76 ± 1.08 (pores) 10.71 ± 2.50 (spheres)	2.9 ± 0.5	6.2 ± 0.7
**MAO 3 (Ca/P ≈ 1.0)**	21.85 ± 5.53	172.5 ± 12.6	0.82 ± 0.20	2.2 ± 0.3	4.7 ± 0.4

Table S2. Wettability results for all investigated samples given by water contact angles (WCA) and diiodmethane contact angles (DCA). The surface energy γ of the samples, as well as their disperse component γ^D and the polar component γ^P of the surface energy.

Sample	WCA (°)	DCA (°)	Surface Energy (mJ/m^2)	Dispersive Component ^{D} (mJ/m^2)	Polar Component ^{P} (mJ/m^2)
**Control**	64.70 ± 4.04	28.40 ± 2.87	53.01 ± 0.99	53.01 ± 0.99	53.01 ± 0.99
**MAO 1**	32.70 ± 4.51	35.40 ± 1.78	63.89 ± 0.87	40.83 ± 0.39	23.06 ± 0.48
**MAO 2**	11.90 ± 2.92	56.40 ± 3.06	71.90 ± 1.20	30.15 ± 0.58	41.75 ± 0.62
**MAO 3**	24.70 ± 2.49	27.10 ± 1.32	72.51 ± 0.64	44.94 ± 0.31	27.58 ± 0.33

Table S3. Elemental composition of the MAO coatings determined by EDX for the elements carbon (C), oxygen (O), aluminium (Al), phosphorus (P), calcium (Ca), titanium (Ti) and the calcium to phosphorus (Ca/P) ratio.

Sample	C (at.%)	O (at.%)	Al (at.%)	P (at.%)	Ca (at.%)	Ti (at.%)	Ca/P
**MAO 1**	3.98 ± 3.54	50.94 ± 1.94	1.07 ± 0.04	9.28 ± 0.40	0.12 ± 0.12	34.61 ± 1.26	–
**MAO 2**	21.10 ± 1.49	44.47 ± 1.31	0.29 ± 0.07	17.13 ± 0.15	6.67 ± 0.18	10.34 ± 0.20	0.39 ± 0.01

**MAO 3**	–	43.92 ± 1.51	0.90 ± 0.15	2.38 ± 0.12	2.49 ± 0.09	50.31 ± 1.36	1.05 ± 0.05

Table S4. The phase composition and crystal lattice parameters of the examined MAO coatings by XRD.

Sample	Phase	Crystal Lattice Parameters	Coherent Scattering Region (Å)	Δd/d×10^{-3}
**MAO 1**	α-Ti	a = 2.9165<br>c = 4.6596	49	1.0
	TiO_2 Rutile	a = 4.5845<br>c = 2.9576	34	1.3
	TiO_2 Anatase	a = 3.7784<br>c = 9.4790	45	6.3
**MAO 2**	α-Ti	a = 2.9203<br>c = 4.6205	64	0.6
**MAO 3**	α-Ti	a = 2.9218<br>c = 4.6651	49	0.8
	TiO_2 Rutile	a = 4.5433<br>c = 2.8612	–	–
	TiO_2 Anatase	a = 3.7883<br>c = 9.4794	45	6.4

Table S5. XRD peak fitting parameters (XC – peak center, W – peak width) calculated from the XRD pattern shown in Figure 3D of the main manuscript.

Sample	Peak Number	XC (2 (°))	W (2 (°))	Area	Area Error
**MAO 1**	1	6.0	11.0	729.8	23.0
	2	25.4	0.5	27.0	2.0
	3	27.6	0.4	18.4	1.8
	4	35.5	0.4	185.7	1.7
	5	18.0	33.0	400.0	4.0
	6	39.5	1.7	97.1	3.9
	7	40.7	0.9	91.7	2.8
	8	48.1	0.5	7.0	0.4
	9	53.5	0.5	9.0	0.5
	10	54.3	0.8	24.7	1.2
	11	57.0	1.0	7.0	0.4
	12	63.0	0.6	8.0	0.4
	13	63.8	0.8	28.0	1.4
	14	69.0	0.5	5.0	0.3

	15	70.0	1.0	6.0	0.3
	16	71.2	0.5	8.0	0.4
	17	75.0	0.5	25.0	1.3
	18	77.0	0.6	12.0	0.6
	19	78.3	0.5	69.0	3.5
**MAO 2**	1	6.0	9.0	589.0	4.4
	2	25.7	13.6	696.0	2.5
	3	35.6	0.5	8.4	0.2
	4	40.3	0.5	5.3	0.3
	5	54.5	20.0	341.0	1.8
	6	75.3	0.8	8.2	0.4
	7	78.0	0.8	19.8	0.4
**MAO 3**	1	7.0	12.0	575.0	28.8
	2	25.0	0.8	39.3	1.4
	3	25.0	12.0	300.0	15.0
	4	35.5	0.5	119.0	6.0
	5	38.6	0.8	30.0	1.5
	6	40.0	0.5	5.0	0.3
	7	40.8	0.5	47.4	1.1
	8	48.1	0.8	20.0	1.5
	9	54.0	5.1	71.4	4.6
	10	55.3	0.5	2.6	1.2
	11	64.0	0.5	5.4	1.3
	12	52.7	2.4	39.0	3.5
	13	71.6	1.5	21.7	19
	14	75.3	0.9	28.9	1.4
	15	77.0	0.5	7.8	1.1
	16	78.3	1.1	63.9	1.7

Table S6. MAO phase composition based on SI Table S4.

Sample	Crystallite Phase Fraction	Area	Area Error	Percentage (%)	Percentage Error
**MAO 1**	Amorphous phase	1129.8	27.0	64.3	1.5
	TiO_2 Anatase	34.0	2.4	1.90	0.1

	TiO_2 Rutile	203.1	10.1	11.5	0.6
	α-Ti	391.4	10.2	22.3	0.6
	Total	1758.3	49.7	100	2.8
**MAO 2**	Amorphous phase	1626.0	8.7	97.5	0.5
	TiO_2 Anatase	–	–	–	–
	TiO_2 Rutile	–	–	–	–
	α-Ti	41.7	1.3	2.5	0.1
	Total	1667.7	10.0	100	0.6
**MAO 3**	Amorphous phase	946.4	48.4	65.4	3.3
	TiO_2 Anatase	59.3	2.9	4.10	0.2
	TiO_2 Rutile	151.4	8.4	10.4	0.6
	α-Ti	290.7	17.1	20.1	1.2
	Total	1447.8	76.8	100	5.3

Table S7. Mechanical properties of the investigated coatings, F_{max} is the maximum indentation force, h_{max} is the maximum indentation height, E_{IT} is indentation elastic modulus and H_{IT} is the indentation hardness.

**Sample**	**F_{max} (mN)**	**h_{max} (m)**	**E_{IT} (MPa)**	**H_{IT} (MPa)**
**MAO 1**	50	0.860 ± 0.155	81400 ± 19486	4255 ± 2061
**MAO 2**	50	4.878 ± 1.966	9620 ± 2928	164 ± 185
**MAO 3**	50	1.018 ± 0.258	52600 ± 19974	3435 ± 1666

Table S8. Calculated parameters of the electrical equivalent circuit elements (EEC, the constant phase elements CPE_1 and CPE_2, the CPE coefficients Q_1 and Q_2) for the electrochemical impedance spectroscopy (EIS) of the MAO 1 – 3 coatings.

**Sample**	***CPE_1***		**R_1 ($\Omega \cdot cm^2$)**	***CPE_2***		**R_2 ($\Omega \cdot cm^2$)**
	**Q_1 ($S \cdot cm^{-2} \cdot s^n$)**	n_1		**Q_2 ($S \cdot cm^{-2} \cdot s^n$)**	n_2	
**Control**	-----	-----	-----	$2.6 \cdot 10^{-5}$	0.94	$2.3 \cdot 10^{6}$
**MAO 1**	$3.5 \cdot 10^{-7}$	0.81	$5.5 \cdot 10^{4}$	$1.2 \cdot 10^{-6}$	0.57	$4.6 \cdot 10^{7}$
**MAO 2**	$2.5 \cdot 10^{-7}$	0.86	$6.7 \cdot 10^{4}$	$5.4 \cdot 10^{-6}$	0.51	$2.0 \cdot 10^{7}$
**MAO 3**	$3.7 \cdot 10^{-7}$	0.86	$5.9 \cdot 10^{4}$	$6.8 \cdot 10^{-6}$	0.51	$5.5 \cdot 10^{6}$

Table S9. Electrochemical parameters (corrosion potential E_C, current density j_C, Tafel slope of the anodic branch of the potentiodynamic polarization curve ${}_a$, Tafel slope of the cathodic branch of the potentiodynamic polarization curve – β_c, polarization resistance Rp and the value of the impedance |Z|) of the MAO 1 – 3 coatings determined by electrochemical impedance spectroscopy (EIS).

Sample	E_C (V) (vs Ag/AgCl)	j_C (A·cm^{-2})	β_a (V/decade)	$-\beta_c$ (V/decade)	R_p, (Ω·cm^2)	$\lvert Z \rvert_{f\to 0\ Hz}$, (Ω·cm^2)
**Ti control**	-0.28	$8.1\cdot10^{-8}$	0.28	0.15	$4.9\cdot10^{5}$	$4.7\cdot10^{5}$
**MAO 1**	0.41	$7.7\cdot10^{-9}$	0.51	0.15	$6.7\cdot10^{6}$	$3.4\cdot10^{6}$
**MAO 2**	0.51	$3.9\cdot10^{-8}$	0.66	0.11	$1.1\cdot10^{6}$	$7.4\cdot10^{5}$
**MAO 3**	0.29	$6.1\cdot10^{-8}$	0.73	0.13	$9.7\cdot10^{5}$	$6.1\cdot10^{5}$

Table S10. Surface coverage rate of cells stained with Alizarine red for the samples control (-) (plastic Petri dish), Ti control (+) (unmodified titanium sample – without a MAO coating) and the MAO 1 – 3 coatings.

Sample	After 21 Days
	Coverage Rate (%)
**Control (-)**	0.000
**Ti control (+)**	0.778
**MAO 1**	7.619
**MAO 2**	0.134
**MAO 3**	18.001

Supplemental References

1. Santiago-Medina, P.; Sundaram, P.A.; Diffoot-Carlo, N. Titanium Oxide: A Bioactive Factor in Osteoblast Differentiation. *Int. J. Dent.* **2015**, *2015*, 1–9, doi:10.1155/2015/357653.
2. Ruan, D.; Wu, C.; Deng, S.; Zhang, Y.; Guan, G. The Anatase Phase of Nanotopography Titania with Higher Roughness Has Better Biocompatibility in Osteoblast Cell Morphology and Proliferation. *Biomed Res. Int.* **2020**, *2020*, 1–8, doi:10.1155/2020/8032718.
3. Chen, H.-T.; Lin, H.-I.; Chung, C.-J.; Tang, C.-H.; He, J.-L. Osseointegrating and Phase-Oriented Micro-Arc-Oxidized Titanium Dioxide Bone Implants. *J. Appl. Biomater. Funct. Mater.* **2021**, *19*, 228080002110068, doi:10.1177/22808000211006878.
4. Yokota, S.; Nishiwaki, N.; Ueda, K.; Narushima, T.; Kawamura, H.; Takahashi, T. Evaluation of Thin Amorphous Calcium Phosphate Coatings on Titanium Dental Implants Deposited Using Magnetron Sputtering. *Implant Dent.* **2014**, *23*, 343–350, doi:10.1097/ID.0000000000000098.
5. Nagano, M.; Nakamura, T.; Kokubo, T.; Tanahashi, M.; Ogawa, M. Differences of Bone Bonding Ability and Degradation Behaviour in Vivo between Amorphous Calcium Phosphate and Highly Crystalline Hydroxyapatite Coating. *Biomaterials* **1996**, *17*, 1771–1777, doi:10.1016/0142-9612(95)00357-6.
